# Human Diversity of Killer Cell Immunoglobulin-Like Receptors and Human Leukocyte Antigen Class I Alleles and Ebola Virus Disease Outcomes

**DOI:** 10.3201/eid2701.202177

**Published:** 2021-01

**Authors:** Tony Wawina-Bokalanga, Bert Vanmechelen, Valentine Lhermitte, Joan Martí-Carreras, Valentijn Vergote, Fara Raymond Koundouno, Joseph Akoi-Boré, Ruth Thom, Tom Tipton, Kimberley Steeds, Kéita Balla Moussa, Ablam Amento, Lies Laenen, Sophie Duraffour, Martin Gabriel, Paula Ruibal, Yper Hall, Mandy Kader-Kondé, Stephan Günther, Guy Baele, Cesar Muñoz-Fontela, Johan Van Weyenbergh, Miles W. Carroll, Piet Maes

**Affiliations:** KU Leuven, Leuven, Belgium (T. Wawina-Bokalanga, B. Vanmechelen, V. Lhermitte, J. Martí-Carreras, V. Vergote, L. Laenen, G. Baele, J. Van Weyenbergh, P. Maes);; University Julius Nyerere of Kankan, Conakry, Guinea (F.R. Koudouno);; Institut National de Santé Publique, Conakry (J. Akoi-Boré);; Public Health England, Salisbury, UK (R. Thom, T. Tipton, K. Steeds, Y. Hall, M.W. Carroll);; Centre d'Excellence de Formation et Recherche sur les Maladies Prioritaires en Guinée, Conakry (K.B. Moussa, A. Amento, M. Kader-Kondé);; German Center for Infection Research, Hamburg-Lübeck-Borstel-Riems, Germany (S. Duraffour, M. Gabriel);; Bernhard Nocht Institute for Tropical Medicine, Hamburg, Germany (S. Duraffour, M. Gabriel, P. Ruibal, S. Günther, C. Muñoz-Fontela)

**Keywords:** killer cell immunoglobulin-like receptor genes, killer cell immunoglobulin-like receptors, human leukocyte antigens class I, human leukocyte antigens, natural killer cells, Zaire ebolavirus, Ebola virus disease, viruses, zoonoses, Ebola virus infection, viral zoonoses, Guinea

## Abstract

We investigated the genetic profiles of killer cell immunoglobulin-like receptors (KIRs) in Ebola virus–infected patients. We studied the relationship between KIR–human leukocyte antigen (HLA) combinations and the clinical outcomes of patients with Ebola virus disease (EVD). We genotyped KIRs and HLA class I alleles using DNA from uninfected controls, EVD survivors, and persons who died of EVD. The activating *2DS4–003* and inhibitory *2DL5* genes were significantly more common among persons who died of EVD; *2DL2* was more common among survivors. We used logistic regression analysis and Bayesian modeling to identify *2DL2*, *2DL5*, *2DS4–003*, *HLA-B-Bw4-Thr*, and *HLA-B-Bw4-Ile* as probably having a significant relationship with disease outcome. Our findings highlight the importance of innate immune response against Ebola virus and show the association between KIRs and the clinical outcome of EVD.

Ebola virus (EBOV) is an enveloped, nonsegmented, negative-sense, single-stranded RNA virus that belongs to the genus *Ebolavirus* in the family *Filoviridae*. This genus comprises 6 species recognized by the International Committee on Taxonomy of Viruses: *Zaire ebolavirus*, *Sudan ebolavirus*, *Bundibugyo ebolavirus*, *Tai Forest ebolavirus*, *Reston ebolaviru*s, and the recently discovered *Bombali ebolavirus* (*1*).

Since the first recorded Ebola virus outbreaks in 1976 in Zaire (now the Democratic Republic of the Congo [DRC]) and southern Sudan, other outbreaks of Ebola virus disease (EVD) have been reported in Africa. The West Africa Ebola virus outbreak in 2013–2016, which mainly affected Guinea, Liberia, and Sierra Leone, was the largest and most widespread EVD outbreak. According to the World Health Organization, this outbreak comprised 28,646 confirmed, probable, and suspected EVD cases and caused 11,323 reported deaths (*2*).

On June 25, 2020, the DRC’s Ministry of Health declared the second largest EVD outbreak to be over (*3*). According to the DRC’s Ministry of Health, this outbreak caused 3,470 confirmed and probable EVD cases. A total of 2,299 infected persons died, and 1,171 persons survived.

EBOV quickly overwhelms the host’s innate immune response and causes an acute febrile illness along with headache, vomiting, abdominal pain, diarrhea, severe fatigue, coagulation disorders, hypotension, lymphopenia, and thrombocytopenia (*4*,*5*). Some EBOV infections generate a cytokine storm, which hinders peripheral natural killer cells (NK) and T and B lymphocytes. This response can induce multiorgan failure, hypovolemic shock, and death (*6*).

NK cells, among other cells, are key effector cells of the innate immune system and play a crucial role in the antiviral response. The effector capability of NK cells has been described in a wide range of viral infections, such as hepatitis B, hepatitis C (HCV), HIV, and human cytomegalovirus infection (*7*). Few studies have examined NK cell levels and function in EVD patients; those studies documented lower NK cell levels among persons who died of EVD compared with survivors (*8*–*10*). One study also observed an increase in inhibitory receptor KIR2DL1 in NK cells during EVD (*8*). In addition, researchers have demonstrated the cytotoxic effect of NK cells during experimentally induced EBOV infection in nonhuman primates and its protective and deleterious effects in NK-depleted mice (*11*–*14*). Activated NK cells respond to EBOV-infected cells by releasing perforin and granzyme, which mediate the cytolysis of EBOV-infected cells. Furthermore, the ability of NK cells to secrete cytokines such as interferon-γ, interferon-α/β, and tumor necrosis factor-α is essential to the immune response (*5*). Reed et al. demonstrated that lethal EBOV infection is associated with loss and decreased activity of NK cells (*11*). In addition, Cimini et al. observed that patients who died of EVD had lower NK cell frequencies than patients who survived (*8*). The mechanism behind this association is still unknown; however, the degree of loss is highly correlated with fatal disease outcome.

The cytotoxic and secretory functions of NK cells are regulated through the interaction between human leukocyte antigens (HLA) class I molecules on target cells and receptors, such as the killer cell immunoglobulin-like receptors (KIRs), on NK cells (*15*). KIRs are members of the immunoglobulin superfamily type I receptors, and they are encoded by a family of highly polymorphic genes located on human chromosome 19q13.4 within the leukocyte receptor complex (*16*). They are expressed on the surface of NK cells and certain T lymphocytes and regulate the function and development of these cells. NK cells recognize HLA class I molecules on the surface of host cells, enabling them to distinguish between self and nonself and to target infected or malignant cells for lysis (*17*).

Researchers have identified 16 KIR genes, of which 8 are inhibitory (*2DL1–5*, *3DL1–3*), 6 are activating (*2DS1–5*, *3DS1*), and 2 are pseudogenes (*2DP1* and *3DP1*). Some KIRs, such as *2DL4*, *3DL2*, and *3DL3*, contain sequences for activating and inhibitory receptors (*18*). Inhibitory KIRs interact with specific motifs of HLA class I molecules. One such motif is the amino acid at position 80 of HLA: lysine or asparagine for HLA-C alleles and threonine or isoleucine for HLA-B-Bw4 and HLA-A-Bw4 alleles (*19*).

The number and types of KIRs vary considerably among individual persons, who might exhibit 7–12 KIRs for each haplotype. A person might have haplotype A, which exhibits more inhibitory KIRs, or haplotype B, which exhibits more activating KIRs (*19*). The AB or BB haplotype is characterized by the presence of the *2DL2*, *2DL5*, *3DS1*, *2DS1*, *2DS2*, *2DS3*, or *2DS5* genes*.* None of these genes are present in the AA haplotype, which contains only a single activating KIR gene, *2DS4* (with a deleted variant of 22 bp in exon 5, *2DS4–003*) (*20*). Because these genes are so variable and the KIR/HLA combinations play a key role in immune response, understanding this variability is important for genotyping studies (*21*). Genetic studies have shown that the distribution of KIR genes and KIR/HLA combinations vary widely; furthermore, these variations can predict disease outcomes in persons with hepatitis B, HCV, human T-lymphotropic virus type 1 (HTLV-1), or HIV-1 infections (*22*,*23*). During HIV-1, HCV, or HTLV-1 infection, CD8+ cells mediate much of the protective effect of inhibitory KIRs (*24*).

We determined the genetic profiles of KIR genes and HLA class I alleles in DNA samples from persons infected with EBOV Makona variant in Guinea. We also assessed the distribution of HLA class I genotypes during an EVD outbreak and the relationship between specific KIR/HLA combinations and the clinical outcomes of persons with EVD.

## Materials and Methods

### Study Samples

We studied samples from patients in whom EVD was diagnosed during the 2013–2016 outbreak. We collected serum samples from 77 uninfected controls and 101 EVD survivors in Guéckédou, Guinea, during May 2015–September 2017. In addition, the European Mobile Laboratory provided DNA isolated from whole blood samples of 119 persons who had died of EVD.

We defined EVD survivors as EBOV-infected patients who had survived the acute phase of EVD and were discharged from the Ebola treatment center in Guéckédou after testing negative for Ebola 4 times by reverse transcription PCR. The survivors in our study included only persons with an original certificate of survivorship issued by the Guinean government. Controls tested negative for EBOV-specific antibodies and EBOV neutralizing antibodies in plasma.

We collected blood samples in EDTA tubes for routine blood tests, serologic assays for EBOV antigen, and nucleic acid detection of EBOV RNA by reverse transcription PCR. Afterward, we isolated peripheral blood mononuclear cells (PBMCs) from whole blood samples using Ficoll-Paque density gradient centrifugation (Sigma-Aldrich Inc., https://www.sigmaaldrich.com) according to the manufacturer’s instructions. To store PBMCs at room temperature, we used DNAgard Blood (Sigma-Aldrich Inc.) according to the manufacturer’s instructions. 

### Genomic DNA Extraction

We extracted genomic DNA from PBMCs from EVD survivors and controls. We used the QIAamp DNA Mini kit (QIAGEN, https://www.qiagen.com) for DNA extraction according to the manufacturer’s instructions. We eluted the purified DNA in 100 μL buffer AE and quantified it using a NanoDrop spectrophotometer (ThermoFisher Scientific, https://www.thermofisher.com).

### Whole-Genome Amplification

We conducted whole-genome amplification of DNA samples using the multiple displacement amplification Repli-g mini kit (QIAGEN) according to the manufacturer’s instructions. We then purified DNA with a rapid in-house method using ethanol precipitation. For this procedure, we first mixed 1:10 volume sodium acetate (3 M, pH 5.5) with 2 volumes absolute ethanol in a 1.5 mL Eppendorf tube. After inverting the tube, we incubated the reaction mixture at −80°C for 2 h and then centrifuged at 20,000 × *g* for 30 min. We withdrew the supernatant and washed the pellet with 70% ethanol, then centrifuged the mixture at maximum speed for 15 min. After removing the supernatant, we air-dried the pellet at 50°C and then resuspended in 50 μL buffer AE. We stored the extracted DNA products at −20°C.

### KIR and HLA Class I Genotyping

We established the presence or absence of 14 KIR genes and 2 pseudogenes (*2DP1* and *3DP1*) by PCR using primers, as published in previous studies (*25*–*27*). We used Primer3 version 0.4.0 software (https://bioinfo.ut.ee/primer3-0.4.0) to design specific primers targeting *2DL5* and *2DS4–001*. We conducted HLA genotyping for 5 HLA class I alleles known to be KIR ligands: *HLA-C1-Asn80* (*2DL2*, *2DL3*, and *2DS2*), *HLA-C2-Lys80* (*2DL1* and *2DS1*), *HLA-B-Bw4-Thr80* (*3DL1*), *HLA-B-Bw4-Ile80* (*3DS1* and *3DL1*), and *HLA-A-Bw4* (*3DL1* and *3DL2*) (*28*) (Appendix Table 1).

We conducted PCR on amplified genomic DNA from uninfected controls, EVD survivors, and persons who died of EVD. We conducted amplifications using a Biometra TRIO-Analytik Jena Thermocycler (Westburg, https://www.westburg.eu). For each PCR, we used a 30 μL mixture containing >10 ng of DNA sample, 6 μL 5× QIAGEN OneStep reverse transcription PCR buffer, 1 μL dNTP mix with 10 mM of each dNTP, 1 μL QIAGEN OneStep reverse transcription PCR enzyme mix, 0.6 μM of each primer, and RNase-free water. In negative samples, we also added 0.6 μM of an internal control primer set to verify the true absence of KIR genes and HLA class I alleles. We ran each PCR in triplicate (Appendix Tables 2, 3). We ran the amplified DNA on 6% polyacrylamide gels and electrophoresed it in 1× Tris-borate-EDTA buffer. We stained gels with Midori green direct and visualized them under UV light using a gel imager camera.

### Statistical Data Analysis

We tested the difference in frequency of KIR/HLA haplotypes using the Fisher exact test. To avoid type I error, we calculated an adjusted p value with Bonferroni correction and used the resulting p value (p<0.02) to determine significance. We conducted binary logistic regression to assess the effect of HLA and KIR genotypes on the clinical outcome of patients with EVD. We selected any variable with a significant univariate test at a relaxed p value of 0.25 as a candidate for the multivariate analysis. This criterion enabled us to reduce the initial number of variables (i.e., genes) in the model, while simultaneously reducing the risk of missing important variables (*29*,*30*). After preselection, we built a binomial logistic regression model that comprised all remaining explanatory variables and performed backward elimination. The model simultaneously used Bayesian information criterion (*31*) and Fisher exact tests at the 5% significance level.

As an extension to the binomial analysis, we conducted multinomial logistic regression. This model assessed the effect of HLA and KIR genotypes on the clinical outcome of patients with EVD and determined the KIR gene profile associated with these outcomes. We conducted Bayesian model averaging (BMA) using the R packages BMA and mlogitBMA (*32*,*33*). These packages enabled us to account for uncertainty about the explanatory variables using a Bayesian information criterion approximation to the posterior model probabilities. After removing variables that generated collinearity issues, we searched the model space using the fast leaps and bounds algorithm (*34*). As the first step in applying BMA to solve the variable selection problem for multinomial logit data, mlogitBMA uses the approach of Begg and Gray (*35*), which approximates large-scale multinomial logistic regressions as a series of binary logistic regressions (*36*).

### Ethics Approval

The National Committee of Ethics in Medical Research of Guinea approved the use of diagnostic leftover samples and corresponding patient data for this study (approval no. 11/CNERS/14). Samples from EVD survivors and controls were collected under ethics protocols approved by the Guinean National Ethics Committee for Research and Health (approval no. 33/CNERS/15). We obtained written and informed consent from controls and EVD survivors. Ethical permission for the work conducted at KU Leuven on the DNA samples was reviewed and approved by the KU Leuven ethics committee under reference no. S58836.

## Results

### KIR Haplotypes Associated with EVD Outcome

**Figure 1:**
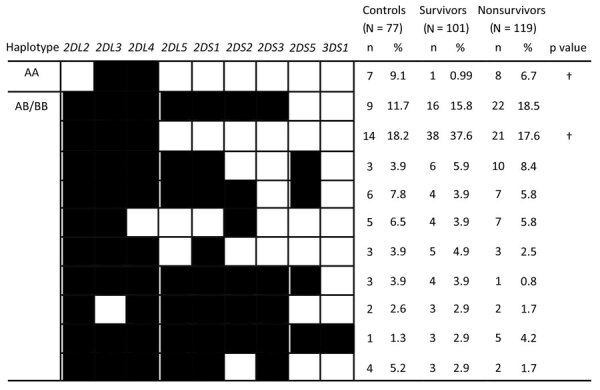
Killer cell immunoglobulin-like receptor haplotypes among the control group, Ebola survivors, and persons who died of Ebola virus disease in Guinea, 2015–2017. Percentage of each haplotype was calculated and defined as the number of persons with the killer cell immunoglobulin-like receptor haplotype (n) divided by the number of persons (N) in the studied group. Black boxes indicate presence of genes; white boxes indicate absence of genes. †p<*Pc* (survivors vs. fatalities). *Pc*, corrected p value.

Of 77 controls, 101 survivors, and 119 persons who died of EVD, an average of 78% of each category had 11 shared haplotypes, which differed from each other by the presence or absence of 9 KIR genes (Figure 1). The remaining participants (20 controls, 14 survivors, and 31 persons who died of EVD) had a rare KIR haplotype that lacked >3 KIR genes (data not shown). The frequency of the KIR AA haplotype was significantly higher for persons who died of EVD (6.7%) than for survivors (0.99%; p = 0.04). One KIR BB haplotype was significantly more common among the survivors (37.6%) than among persons who died of EVD (17.6%; p<0.01). All participants had the *2DL1*, *3DL1*, *3DL2*, *3DL3*, *2DP1*, and *3DP1–004* genes.

### Frequency of KIR Genotypes

We found the *2DL2* gene in 98% of survivors and 83.2% of persons who died of EVD. Likewise, we found the *2DL5* gene in 46.5% of survivors and 63% of persons who died of EVD (Table 1). These results suggest that inhibitory *2DL2* and *2DL5* might be associated with the outcome of persons with EVD.

**Table 1:** Frequency of KIR genes and HLA class I alleles among control group, Ebola survivors, and persons who died of Ebola virus disease, Guinea, 2015–2017*

Gene	Controls, no. (%)	Survivors, no. (%)	Persons who died, no. (%)	p value
Inhibitory KIRs				
* 2DL1*	77 (100.0)	101 (100.0)	119 (100.0)	
* 2DL2*	67 (87.0)	99 (98.0)	99 (83.2)	†‡
* 2DL3*	71 (92.2)	94 (93.1)	112 (94.1)	
* 2DL4*	77 (100.0)	100 (99.0)	118 (99.1)	
* 2DL5*	42 (54.5)	47 (46.5)	75 (63.0)	†
* 3DL1*	77 (100.0)	101 (100.0)	118 (99.1)	
* 3DL2*	76 (98.7)	101 (100.0)	119 (100.0)	
* 3DL3*	77 (100.0)	101 (100.0)	119 (100.0)	
Activating KIRs				
* 2DS1*	46 (59.7)	55 (54.4)	74 (62.2)	
* 2DS2*	37 (48.0)	46 (45.5)	55 (46.2)	
* 2DS3*	23 (29.8)	30 (29.7)	40 (33.6)	
* 2DS4–001*	70 (90.9)	94 (93.1)	103 (86.6)	
* 2DS4–003*	40 (51.9)	41 (40.6)	67 (56.3)	†
* 2DS5*	23 (29.8)	26 (25.7)	40 (33.6)	
* 3DS1*	5 (6.5)	4 (3.9)	13 (10.9)	
Pseudogenes				
* 2DP1*	77 (100.0)	100 (99.0)	116 (97.5)	
* 3DP1–001*	6 (7.8)	4 (3.9)	8 (6.7)	
* 3DP1–004*	76 (98.7)	99 (98.0)	117 (98.3)	
HLA class I alleles				
HLA-C1^Asn80^	55 (71.4)	78 (77.2)	85 (71.4)	
HLA-C2^Lys80^	58 (75.3)	74 (73.3)	88 (73.9)	
HLA-B-Bw4^Thr80^	8 (10.4)	9 (8.9)	22 (18.5)	
HLA-B-Bw4^Ile80^	33 (42.8)	48 (47.5)	38 (31.9)	
HLA-A-Bw4	23 (29.8)	26 (25.7)	37 (31.1)	

*Frequency (%) of each KIR gene or HLA class I allele was calculated and defined as the number of individuals having the gene or allele divided by the number of persons in the studied group. HLA, human leukocyte antigen; KIR, killer cell immunoglobulin-like receptor; *Pc*, corrected p value. †p value <*Pc* (survivors vs. persons who died). ‡p value <*Pc* (controls vs. survivors).

### Relevant KIR Genes and HLA Class I Alleles in Survivors and Persons Who Died of EVD

The binomial logistic regression model (Table 2) retained 4 KIR genes and 2 HLA class I alleles as covariates in our final model, implying that these genes exhibit a significant relationship with disease outcome. Because of the relatively small number of participants, we validated these findings through BMA. The KIR genes *2DL2*, *2DL5*, and *2DS4–0003* and the HLA class I alleles *HLA-B-Bw4-Thr* and *HLA-B-Bw4-Ile* were present in >15% of the models explored during our BMA analysis (Table 3; Appendix Figure 1). These 5 covariates are almost identical to those identified in the binary regression models (Tables 2, 3).

**Table 2:** Results from multivariate logistic regression model of human leukocyte antigen class I and killer cell immunoglobulin-like receptor genes in controls and Ebola virus–infected patients, Guinea, 2015–2017

Term	β estimate	SE	p value
Intercept (β0)	−2.27	0.84	<0.01
*2DL2* (β1)	2.73	0.82	<0.01
*2DL5* (β2)	−1.94	0.67	<0.01
*2DS1* (β3)	1.35	0.67	<0.05
*2DS4–0003* (β4)	−0.63	0.31	<0.05
*HLA-B-Bw4-Thr* (β5)	−1.20	0.48	<0.05
*HLA-B-Bw4-Ile* (β6)	0.76	0.31	<0.05

**Table 3:** Results from the multinomial logistic regression of human leukocyte antigen class I and killer cell immunoglobulin-like receptor genes in Ebola virus–infected patients, Guinea, 2015–2017*

Term	p ≠ 0	β estimate	SE	Best model
*2DL2*	88.1	1.02	0.54	1.18
*2DL5*	59.1	−0.43	0.46	−0.65
*KIR2DS4.0003*	16.6	−0.08	0.20	(...)
*HLA-B-Bw4-Thr*	45.8	−0.38	0.48	(...)
*HLA-B-Bw4-Ile*	45.1	0.27	0.34	(...)

*Intercepts have been omitted for interpretation purposes. p ≠ 0 indicates the probability that the coefficient for a given predictor is not zero. Ellipses (...) represent predictors that were not present in the best model.

### HLA Haplotypes

**Figure 2:**
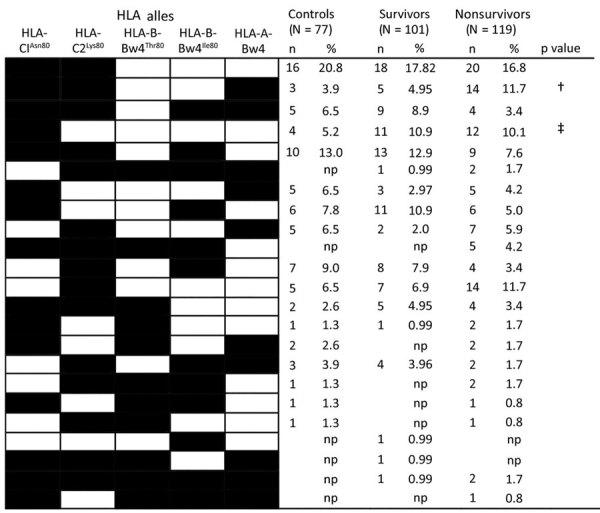
HLA haplotypes identified among the control group, Ebola survivors, and persons who died of Ebola virus disease in Guinea, 2015–2017. Percentage of each haplotype was calculated and defined as the number of persons with the HLA haplotype (n) divided by the number of individuals (N) in the studied group. Black boxes indicate presence of genes; white boxes indicate absence of genes. †p<*P*c (survivors vs fatalities); ‡p<*Pc* (controls vs infected cases). HLA, human leukocyte antigen; NP, not present; *Pc*, corrected p value.

We identified 23 HLA class I haplotypes. These haplotypes differed by the presence or absence of 2 HLA-C, 2 HLA-B, or 1 HLA-A alleles (Figure 2). The HLA haplotype characterized by the presence of *HLA-C1^Asn80^*, *HLA-C2 ^Lys80^*, and *HLA-A-Bw4* was significantly more common among persons who died (11.7%) than survivors (4.95%; p<0.01).

### Functional Analysis of HLA and KIR Gene Combinations

**Figure 3:**
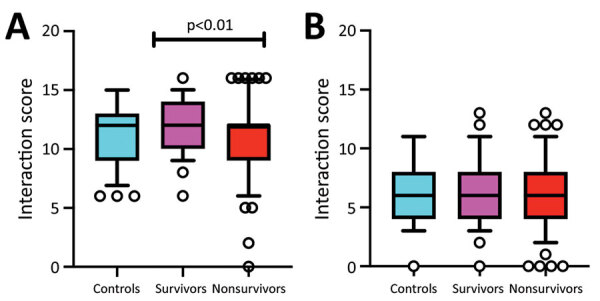
Statistical comparison of all inhibitory and activating killer cell immunoglobulin receptors (KIRs) between controls, survivors, and persons who died of Ebola virus disease in Guinea, 2015–2017. A) All inhibitory KIRs with their specific HLA ligands are compared between studied groups. Persons who did and did not survive differed significantly. B) Comparison of activating KIRs with their specific HLA ligands between studied groups. HLA, human leukocyte antigen.

To further evaluate whether biologically relevant KIR/HLA combinations affect the outcome of patients with EVD, we conducted a functional analysis of 5 HLA class I alleles and their respective inhibitory and activating KIRs (Appendix Table 1); we compared the results of uninfected controls, persons who died of EVD, and survivors. We used previously validated functional KIR/HLA pairs (*28*) to stratify each group of participants into inhibitory and activating KIR/HLA ligands and scored each KIR/HLA combination: 0, absence of both KIR and HLA ligand; 1, presence of either KIR or HLA ligand; 2, weak affinity between KIR and HLA; or 3, strong affinity between KIR and HLA. Survivors had a total inhibitory KIR/HLA score that was significantly higher than in persons who died of EVD (p<0.01 by Kruskall-Wallis test with false discovery rate correction), whereas no significant difference was observed in the total activating KIR/HLA score Figure 3.

## Discussion

Despite the presumed protective role played by NK cells against EBOV, few studies have been conducted on KIRs and their specific HLA ligands during EVD (*6*,*37*). Our study shows that EBOV-infected patients have diverse HLA and KIR genotypes. The KIR haplotype lacking the *2DL2*, *2DL5*, *2DS1*, *2DS2*, *2DS3*, *2DS5*, and *3DS1* genes was significantly more common among persons who died of EVD (6.7%) than survivors (0.99%; p = 0.04) (Figure 1). We used BMA analysis to identify the KIR genes *2DL2*, *2DL5*, and *2DS4–003* as possibly associated with disease outcomes. Contrary to findings by Wauquier et al. (*37*), we found that frequencies of activating *2DS1* and *2DS3* were not significantly correlated with disease outcomes. In addition, the KIR *2DS4–003* and *2DL5* genes were significantly more common among persons who died of EVD than among survivors. This discrepancy might be caused by differences in sample size or genetic makeup of the study populations.

Using the KIR Allele Frequency Net Database (http://www.allelefrequencies.net), we compared the frequency of inhibitory and activating KIR genes from this study with different populations. These populations were from countries in West Africa (i.e., Côte d’Ivoire, Nigeria, Ghana, Equatorial Guinea, and Senegal), Central Africa (i.e., DRC, Gabon, and Uganda), South Africa (i.e., South Africa and Zimbabwe), and a group of mixed population from Reunion, Comoros, and South Africa. Inhibitory *2DL2* and activating *2DS1* frequencies were higher for our study group than for all other studied populations from West Africa (p<0.01), Central Africa (p<0.01), and South Africa (p<0.01) (Appendix Figure 2). The apparent discrepancy between our study and that of Wauquier et al. (*37*) might be largely explained by differences in population genetics between Guinea and Gabon, where Wauquier et al. recruited participants. These differences might be promulgated by the rapid evolution of human KIR genes, partly in response to viral diversity (*38*).

Another study found increased levels of inhibitory KIR *2DL1* at the cell surface of NK cells in EVD patients from Guinea (*8*) during the same outbreak. However, that study’s small sample size did not enable the detection of differences between the 8 survivors and 6 persons who died of EVD. Our data suggests that KIR *2DL2* has a protective role in EVD; similarly, this gene is protective against HIV-1 and HCV infection because it enhances the protective effect of HLA-B57 on viral load, slows the reduction in CD4 count, and enables spontaneous clearance of HCV (*23*). On the other hand, KIR *2DL2* strongly enhanced the protective effect of HLA-C8 and the detrimental effect of HLA-B54 on disease outcome in HTLV-1 infection (*24*). It will be important to further investigate whether the proposed protective effect of KIR *2DL2* in EVD might be mediated by the patient’s viral load.

All participants in our study had the KIR *3DL3* gene. Existing data show that *3DL3* transcripts are overexpressed in persons who died of EVD compared with survivors in recovery (p<0.01) and in the viremic phase (p = 0.17; data not shown) (*39*). Our findings support the hypothesis that genomic and transcriptomic mechanisms of KIR regulation play a role in EVD outcomes.

Although persons from all groups had the inhibitory *3DL1* gene, the frequency of its low-affinity ligand HLA-B-Bw4^Thr80^ was significantly higher among persons who died (18.5%) than among survivors (8.9%). In contrast, its high-affinity ligand HLA-B-Bw4^Ile80^ allele was more common among survivors (47.5%) than among persons who died of EVD (31.9%).

In conclusion, EVD survivors express less activating and more inhibitory KIRs (Table 2) and more functional inhibitory KIR/HLA pairs (Figure 3) through genomic and transcriptomic (*39*) mechanisms, than persons who died of EVD. We hypothesize that these genetic differences contribute to the uncontrolled innate immune response observed in EVD (*6*). This response is mediated mostly by NK cells, although KIRs might also participate (*24*).

Although researchers have made substantial advances in drug and vaccine development for EVD in the last 5 years, researchers should also investigate the potential effects of blocking KIR receptors on disease outcome. These biomarkers could lead to new therapeutic approaches, preferentially targeting the innate immune system, for future EVD outbreaks. Our study had a reasonable sample size, but further investigations should examine a larger cohort. 

Appendix Additional information on primer sequences and PCR conditions for killer cell immunoglobulin-like receptors and human leukocyte antigen class I alleles.
