# Supplementary material for: Human Diversity of Killer Cell Immunoglobulin-Like Receptors and Human Leukocyte Antigen Class I Alleles and Ebola Virus Disease Outcomes
Source: Emerg Infect Dis. 2021 Jan;27(1):76–84. doi: 10.3201/eid2701.202177 (PMC7774578; doi:10.3201/eid2701.202177)
Supplement: Appendix — Additional information on primer sequences and PCR conditions for killer cell immunoglobulin-like receptors and human leukocyte antigen class I alleles. [file 20-2177-Techapp-s1.pdf]

Article DOI: https://doi.org/10.3201/eid2701.202177

Human Diversity of Killer Cell Immunoglobulin-Like Receptors and Human Leukocyte Antigen Class I Alleles and Ebola Virus Disease Outcomes

Appendix

Appendix Table 1. Primer sequences of KIR genes and HLA class I alleles*

Gene	Forward primer (5′-3′)	Reverse primer (5′-3′)	Size, bp
KIR genes			
2DL1	TTGGTCAGATGTCATGTTTGAA	TCCCTGCCAGGTCTTGCG	143
2DL2	AAACCTTCTCTCTCAGCCCA	GCCCTGCAGAGAACCTACA	142
2DL3	ACAAGACCCTCAGGAGGTGA	GCAGGAGACAACTTTGGATCA	160
2DL4	TCAGGACAAGCCCTTCTGC	GACAGGGACCCCATCTTTC	130
2DL5	GCGCTGTGGTGCCTCG	GACCACTCAATGGGGGAGC	214
2DS1	GTAGGCTCCCTGCAGGGA	ACAAGCAGTGGGTCACTTGAC	148
2DS2	CTGCACAGAGAGGGGAAGTA	CAGAGGGTCACTGGGAGC	177
2DS3	ACCTTGTCCTGCAGCTCCT	AGCATCTGTAGGTTCCTCCT	160
2DS4–001	CTGGCCCTCCCAGGTCA	TCTGTAGGTTCCTGAAAGGACAG	204
2DS4–003	CTTGTCCTGCAGCTCCATC	TGACGGAAACAAGCAGTGGA	202
2DS5	TGATGGGGTCTCCAAGGG	TCCAGAGGGTCACTGGGC	105
3DL1	TGAGCACTTCTTTCTGCACAA	TAGGTCCCTGCAAGGGCAA	129
3DL2	AAACCCTTCCTGTCTGCCC	TGGAAGATGGGAACGTGGC	134
3DL3	GCAATGTTGGTCAGATGTCAG	AGCCGACAACTCATAGGGTA	199
3DS1	TCCATCGGTTCCATGATGCG	GACCACGATGTCCAGGGGA	111
2DP1	ACATGTGATTCTTCGGTGTCAT	GTGAACCCCGACATCTGTAC	167
3DP1–001	GGTGTGGTAGGAGCCTTAG	GAAAACGGTGTTTCGGAATAC	280
3DP1–004	CGTCACCCTCCCATGATGTA	GAAAACGGTGTTTCGGAATAC	395
HLA class I alleles			
HLA-C1 ^{Asn80}	GAGGTGCCCGCCCGGCGA	CGCGCAGGTTCCGCAGGC	332
HLA-C2 ^{Lys80}	GAGGTGCCCGCCCGGCGA	CGCGCAGTTTCCGCAGGT	332
HLA-A-Bw4	TGGCGCCCCGAACCCTCG	GCTCTGGTTGTAGTAGCGGA	456
HLA-B-Bw4 ^{Thr80}	GGAGCGAGGGGACCGCAG	GTAGTAGCGGAGCGCGGTG	344
HLA-B-Bw4 ^{Ile80}	GAGCGAGGGGACCGCAG	GTAGTAGCGGAGCGCGATC	343

*HLA, human leukocyte antigen; KIR, killer cell immunoglobulin-like receptor (*1*).

Appendix Table 2. PCR conditions of human leukocyte antigen class I and killer cell immunoglobulin-like receptor primer set (except *2DS3*, *2DL5* and *3DP1–001*)*

No. cycles	Stage	Temperature, °C	Time, min
NA	Initial denaturation	94	5.00
NA	Denaturation	94	0.50
10	Annealing	58	0.75
NA	Elongation	72	1.00
NA	Denaturation	94	0.50
20	Annealing	55	0.75
Not applicable	Elongation	72	1.00
Not applicable	Final extension	72	10.00

Appendix Table 3. PCR conditions of *2DS3*, *2DL5* and *3DP1–001* killer cell immunoglobulin-like receptor genes

No. cycles	Stage	Temperature, °C	Time, min
NA	Initial denaturation	94	5.00
NA	Denaturation	94	0.50
35	Annealing	55	0.75
Not applicable	Elongation	72	1.00
Not applicable	Final extension	72	10.00

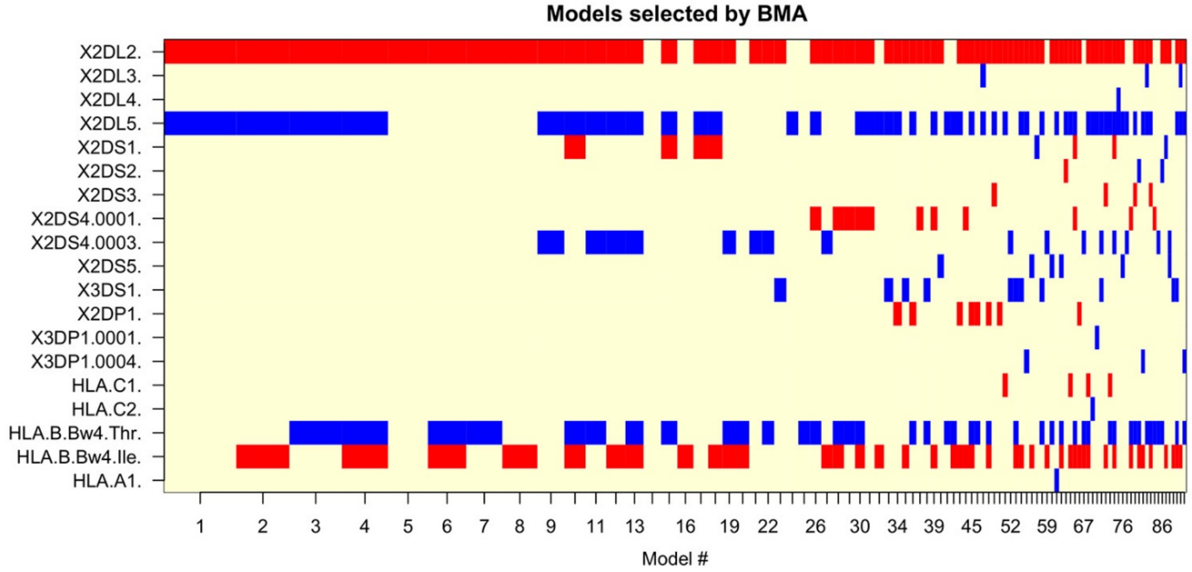

Appendix Figure 1. BMA analysis for multinomial logistic regression of killer cell immunoglobulin-like receptors and human leukocyte antigen class I alleles. Red indicates models with a positive coefficient; blue indicates models with a negative coefficient. Selected variables (genes) shown on the vertical axis; BMA-selected models shown on the horizontal axis. BMA, Bayesian model averaging.

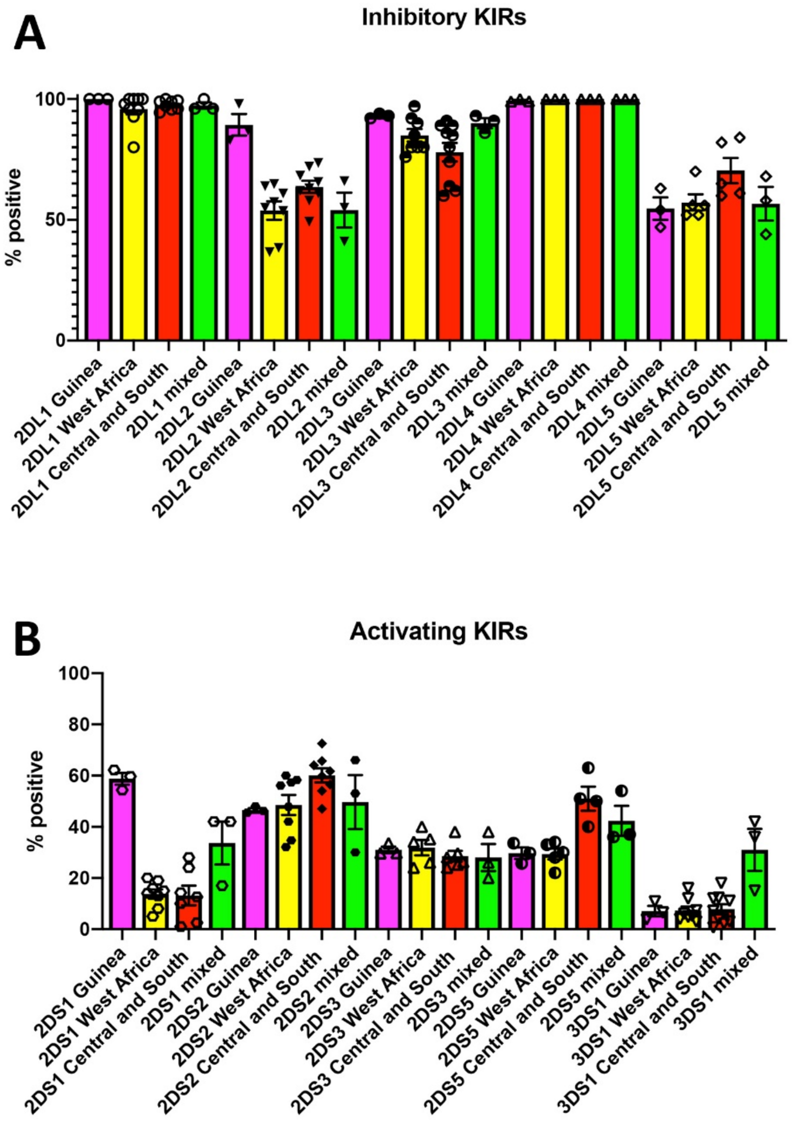

Appendix Figure 2. Comparison of KIR gene frequency in selected populations from countries in West Africa (Ivory coast, Nigeria, Ghana, Equatorial Guinea, and Senegal), Central Africa (the Democratic Republic of Congo, Gabon, and Uganda), South Africa (South Africa, and Zimbabwe), and a mixed population from Reunion, Comoros, and South Africa. A) Inhibitory KIRs. B) Activating KIRs. Data from http://www.allelefrequencies.net. KIR, killer cell immunoglobulin-like receptor.

Reference

1. Tajik N, Shahsavar F, Nasiri M, Radjabzadeh MF. Compound KIR-HLA genotype analyses in the Iranian population by a novel PCR-SSP assay. Int J Immunogenet. 2010;37:159–68. PubMed https://doi.org/10.1111/j.1744-313X.2010.00906.x
